# Investigation of surface subsidence behaviour in multi-slice longwall mining using numerical simulations in Barapukuria coal mine, Bangladesh

**DOI:** 10.1038/s41598-025-31680-0

**Published:** 2026-01-10

**Authors:** Chunlei Zhang, Arifuggaman Arif, Jingke Wu, Md Habibullah, Khezr Mohammadamini, Mahabub Hasan Sajib, Boyina Manohar

**Affiliations:** 1 Faculty of Architecture and Civil Engineering, Huaiyin Institute of Technology, Huai’an, 223001 Jiangsu China; 2 School of Intelligent Manufacturing and Smart Transportation, Suzhou City University, Suzhou, 215104 China; 3 School of Mines, China University of Mining and Technology, Xuzhou, 221116 Jiangsu China; 4 School of Mining Engineering, University of Tehran, Tehran, Iran; 5 Department of Civil Engineering, National Institute of Technology Warangal, Warangal, 506004 India

**Keywords:** Surface subsidence, Ground subsidence, FLAC3D, Numerical modelling, Longwall mining, Stress redistribution, Engineering, Environmental sciences, Natural hazards, Solid Earth sciences

## Abstract

This study investigates surface subsidence induced by multi-slice longwall mining in the Barapukuria coal basin using FLAC3D numerical simulations. The research quantifies the progressive vertical and horizontal deformations caused by mining over multiple phases, analysing the redistribution of stress in the surrounding strata and the corresponding surface displacements. The results demonstrate a clear nonlinear escalation of vertical subsidence, with maximum displacements reaching 5.14 m after the third mining slice. Horizontal displacement similarly increased, with peak values reaching 1.91 m. The study reveals that the subsidence ratio, defined as vertical displacement relative to mining thickness, increased from 0.08 to 0.86 as mining depth progressed, while the horizontal deformation coefficient decreased. Stress redistribution analysis shows that the peak vertical stress in the overburden reached 35.50 MPa in the third phase, leading to significant compaction and fracturing of the strata, which contributed to the observed subsidence. These findings underscore the cumulative and nonlinear nature of surface deformation, emphasizing the critical need for advanced subsidence prediction models and effective mitigation strategies. In particular, the research offers practical insights into managing surface displacement in areas with dense infrastructure, such as residential, industrial, and transportation networks, as well as in environmentally sensitive regions. The study further highlights the importance of incorporating stress redistribution mechanisms to enhance subsidence management, thereby minimizing the risk of damage to local infrastructure and ensuring sustainable mining practices.

## Introduction

**Fig. 1:**
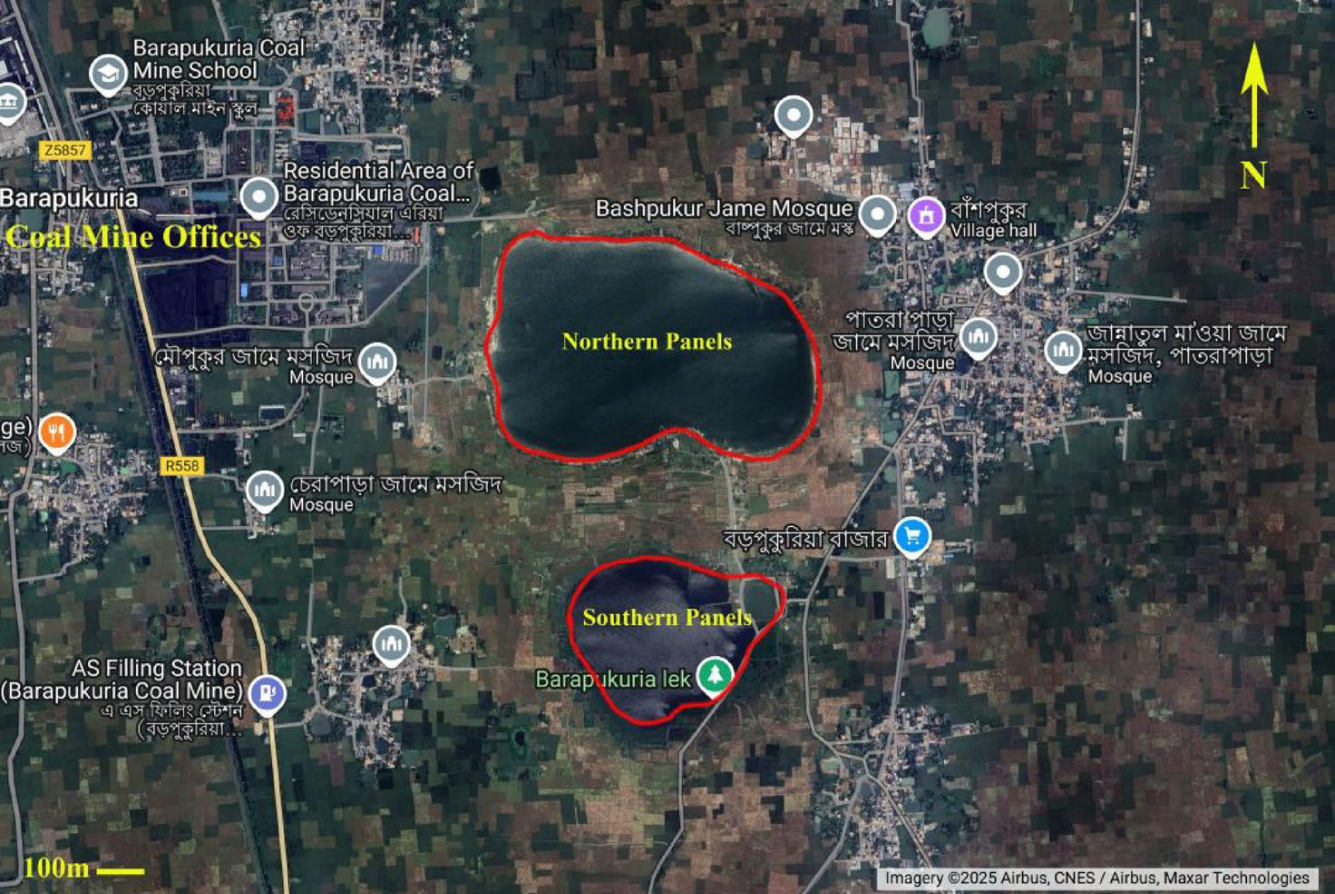
Subsidence zones in the Barapukuria coal mine area. (Google Maps^8^).

Subsidence is an inherent aspect of the longwall coal mining method, which is characterized by lower incidences of stress-related accidents and comparatively higher production efficiency. In the absence of backfilling (stowing), subsidence becomes inevitable in longwall operations, making accurate prediction of mining-induced subsidence a critical component for sustainable mine planning and management^1–3^. The extraction process in longwall mining can trigger various forms of ground movement, including vertical displacement, surface curvature (tilt), and horizontal ground strain (extensional strain)^4^. The severity of damage to surface structures and infrastructure depends largely on their relative location to the subsidence zone^5^. In the Barapukuria coal mining area of Bangladesh, surface subsidence was first documented in 2006, evidenced by visible cracks in overlying structures. Consequently, the government acquired approximately 2.61 km^2^ of the impacted land^6,7^. Aerial imagery of the Barapukuria mining zone reveals that the subsided area can be distinctly divided into northern and southern regions based on the locations of the subsidence epicentres, as illustrated in Fig. 1^8^.

The northern subsidence region can be further divided into two distinct zones: the North-Western and North-Eastern areas. Maximum vertical displacements in these zones are recorded at 5.8 m and 4.6 m, respectively, while the southern region exhibits a peak subsidence of 4.2 m^3^. Recent data indicate that coal mining activities have caused subsidence over approximately 585 acres of land, with deformation magnitudes ranging from as little as 0.01 m up to 10.5 m^9^.

Various methods have been developed to predict mining-induced subsidence, broadly categorized into empirical, semi-empirical, and numerical approaches^10^. Empirical methods, which depend largely on historical subsidence records and pre-defined mathematical models, are relatively simple and cost-effective-particularly in regions with extensive mining history^11^. Nonetheless, they present several limitations. A major shortcoming lies in their dependence on historical datasets, which may be incomplete, outdated, or unrepresentative of present geological conditions, particularly in geologically complex or less-explored areas. These models also exhibit limited adaptability to advancements in mining technologies, evolving extraction techniques, or shifting geological dynamics, often resulting in reduced predictive accuracy over time. In settings characterized by irregular mining patterns, such as non-standard layouts or variable seam depths, empirical approaches may overlook critical influencing factors. Moreover, these models typically assume simplified geological settings, which limits their applicability in zones with complex fault systems, heterogeneous lithology, or unpredictable subsurface behaviour, thereby undermining the reliability of their forecasts^12–16^.

In light of the intricate geological conditions and the multitude of variables influencing subsurface behaviour during mining operations, numerical simulation has become an indispensable method for modelling both underground rock mass response and surface deformation. The advancement of high-performance computing has significantly enhanced the applicability of such simulations. Various techniques have been applied in the prediction of mining-induced subsidence, including the Finite Element Method (FEM)^17^, the Distinct Element Method (DEM)^18^, the Finite Difference Method (FDM)^19,20^, physical modelling approaches^21,22^, as well as geospatial tools such as Geographic Information Systems (GIS) and remote sensing technologies^23^. Among the commonly utilized software platforms, FLAC (FDM-based), MIDAS, Abaqus, and ANSYS (all FEM-based) are prominent. Of these, FLAC3D is particularly advantageous for simulating large-scale deformations in goaf zones due to its capacity to accommodate complex, non-linear, and elasto-plastic material behaviour. Utilizing FLAC3D, Shi et al. examined the temporal evolution of stress and deformation associated with coal seam extraction^24^. Similarly, Hu et al. conducted a comprehensive three-dimensional numerical investigation of the goaf in the Jinjie coal mine, focusing on surface deformation characteristics such as horizontal displacement, vertical settlement, extensional strain, and surface tilt—providing a theoretical framework for managing ground movement in post-mining areas^25^. Additionally, Li et al. combined FLAC3D modelling with graphical techniques to elucidate the underlying mechanisms of surface subsidence and assess the influence of tectonic features, such as faults and seismic activity, on deformation patterns^26^.

Accurate selection of rock mechanical parameters is critical for the reliability of numerical simulations. In practice, these parameters are typically derived from laboratory experiments or in-situ field tests. However, during the initial phases of geological exploration, many mining sites lack sufficient data, particularly with respect to the mechanical properties of overlying strata. In such cases, the absence of experimentally validated input parameters can significantly compromise the fidelity of simulation outcomes, leading to reduced predictive accuracy in modelling subsidence behaviour.

This study aims to fill the existing gap in understanding the surface subsidence induced by multi-slice longwall mining in the Barapukuria coal basin. While previous research has primarily focused on surface displacement and stress redistribution in mining regions, a detailed, phase-by-phase analysis of both vertical and horizontal deformation in relation to varying geological conditions remains limited. By employing FLAC3D simulations, this research seeks to provide a more accurate subsidence prediction model, assess the cumulative impact of mining phases, and evaluate the role of stress redistribution in influencing surface deformation. Additionally, the study proposes effective mitigation strategies that address the challenges posed by subsidence, aiming to safeguard infrastructure and reduce environmental impacts in mining-affected areas.

## Study area

### Geological settings

**Fig. 2:**
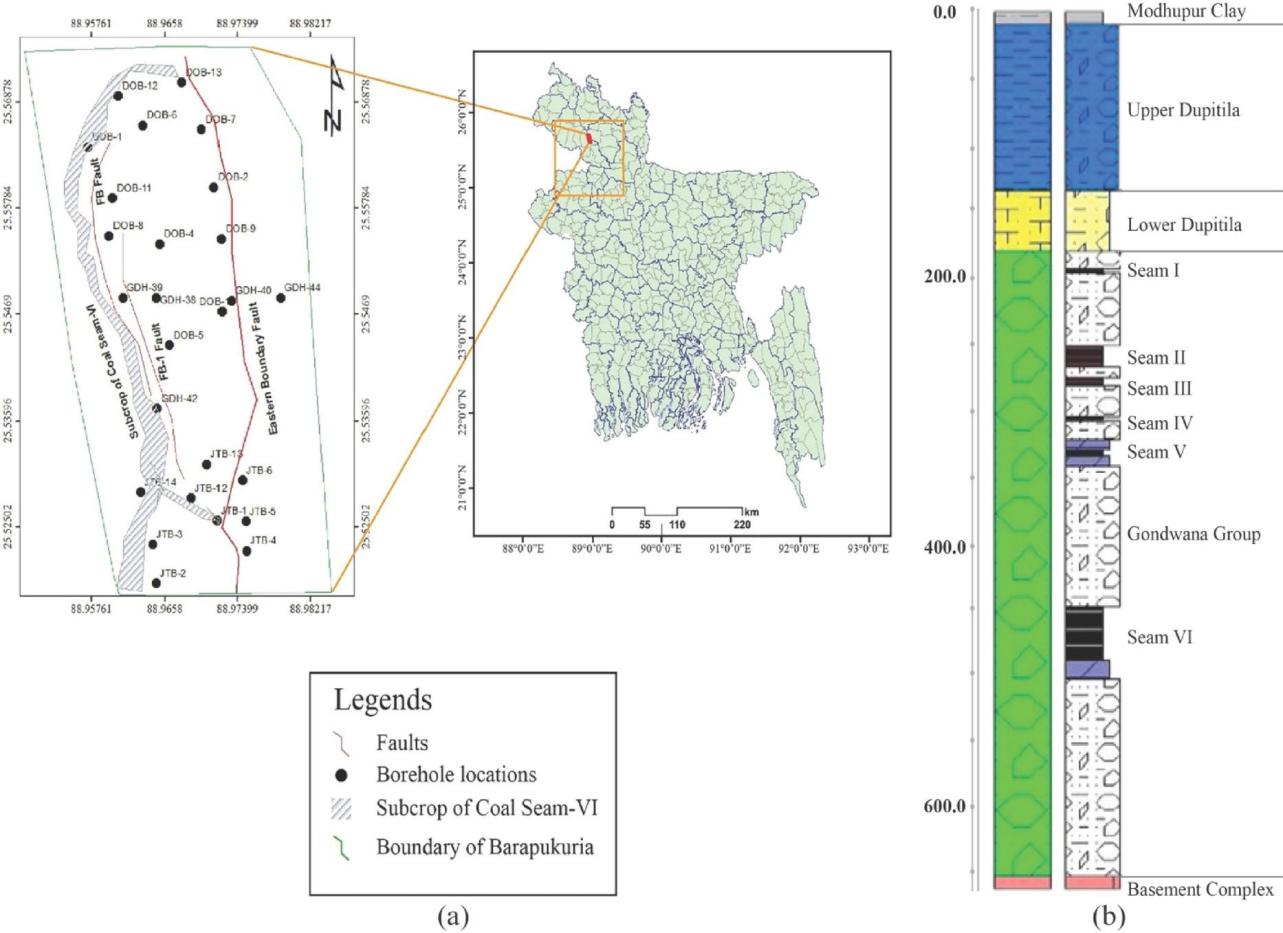
Geographic location and stratigraphic framework of the Barapukuria coal field. (**a**) Geographic location of the Barapukuria coal basin in Bangladesh^30^, (**b**) Stratigraphic profile of the Barapukuria coal basin^31^.

The Barapukuria coal basin is situated in Parbatipur, approximately 50 km^2^ east of Dinajpur district, within the northwestern region of Bangladesh, encompassing an area of 6.68 km^2^^27^. Geographically, it extends between latitudes 25 °32′ 10′′ N to 25 °33′ 20′′ N and longitudes 88 °58′ 00′′ E to 88 °58′ 30′′ E (Fig. 2). The basin lies at a relatively low elevation, ranging from + 30 to 32 m above mean sea level, and is characterized by a predominantly flat surface topography^28^. On a regional scale, the terrain exhibits a gentle southerly gradient and is traversed by tributaries of the Jamuna River^29^. Figure 2 illustrates the geographic setting of the Barapukuria coalfield along with its stratigraphic profile showing the sequence of coal seams and surrounding geological formations in the Barapukuria coal basin**.**

The Barapukuria coal basin is structurally classified as a graben—an asymmetrical, fault-bounded synclinal depression—aligned roughly along a north–south axis^32^. The stratigraphic succession within the basin comprises five principal lithostratigraphic units: (1) Madhupur Clay Formation (MC), (2) Upper Dupi Tila Formation (UDT), (3) Lower Dupi Tila Formation (LDT), (4) Gondwana Formation (GW), and (5) Basement Complex (BC).

The MC, ranging in age from the Holocene to the present, has a thickness of approximately 1 to 15 m across the basin. Beneath the MC lies the Dupi Tila (DT) sequence, primarily of Late Miocene to Middle Pliocene age. The UDT is composed predominantly of unconsolidated to semi-consolidated sand, characterized by medium to coarse grain size, occasional gravel content, and interbedded silt layers. Its thickness ranges from 94 to 126 m within the basin, averaging around 100 m in the mining area. The LDT comprises sandstone, silt, and white clay, with a thickness varying from 0 to 80 m in the basin, and 0 to 60 m within the mine site. Underlying the DT sequence is the GW, a Permian-aged coal-bearing unit that rests unconformably atop the Basement Complex. The GW reaches up to 390 m in thickness across the basin and ranges from approximately 150 to 300 m in the mine area (Fig. 2b). It consists primarily of arkosic sandstones, along with subordinate siltstones, shales, and breccia-conglomerates, interspersed with occasional siltstone and sandstone layers^29,32,33^. The coal seams, including the principal seam of economic interest, are hosted within this formation. The thickest seam attains an average thickness of approximately 36 m and dips gently eastward at an angle of 13° to 19°. The underlying BC is composed predominantly of crystalline rocks, including diorite, meta-diorite, ophiolitic gneiss, and granite^29,33^.

The western segment of the Barapukuria coal basin exhibits a higher degree of faulting compared to its southern counterpart (Fig. 2a). Structurally, the basin is bounded on the east by the Eastern Boundary Fault (EBF) and on the west by a series of subsidiary faults. The fault system within the basin can be broadly categorized into two types: (i) intra-basinal faults and (ii) boundary faults. The EBF dips steeply westward at an angle of approximately 70–75°, with a vertical displacement of nearly 200 m. It extends over a length of about 5 km and exhibits a dominant strike orientation of NNW-SSE to N-S. Western faults primarily follow a NNW-SSE trend, with localized variations trending NNE–SSW^3,29,32,33^. Several intra-basinal faults are present within the coal-bearing strata in the mining area, each exhibiting vertical displacements of roughly 10 m. Additionally, a dyke-an igneous intrusive body-has been identified within the northern mining panels, with a strike orientation trending approximately NEE–SWW^3,28,29^.

### Mining method

Approximately 70–80% of thick coal seams worldwide are extracted using underground mining techniques^34,35^. Among these, longwall and room-and-pillar methods are the two most widely employed for exploiting relatively flat-lying coalbeds^36^. The longwall method involves delineating a coal seam into panels of defined width, length, and height by developing surrounding access roadways. It is a highly mechanized and continuous operation, utilizing self-advancing hydraulic roof supports, a shearer or coal-cutting machine, and an armoured face conveyor aligned parallel to the coal face. In contrast, the room-and-pillar method follows a stepwise, cyclic process wherein coal is extracted from “rooms,” leaving behind “pillars” of unmined coal to support the roof.

An alternative technique, Longwall Top Coal Caving (LTCC), has been effectively applied in China since the mid-1980s, and is now preferred in several other countries, including Australia and Turkey^37^. Compared to multi-slice mining, LTCC is more efficient for thick seams due to its simplified layout and reduced development requirements, resulting in higher production rates. Conversely, multi-slice longwall mining, while technically feasible, is less efficient and more labour-intensive for thick seam conditions^38–41^.

**Fig. 3:**
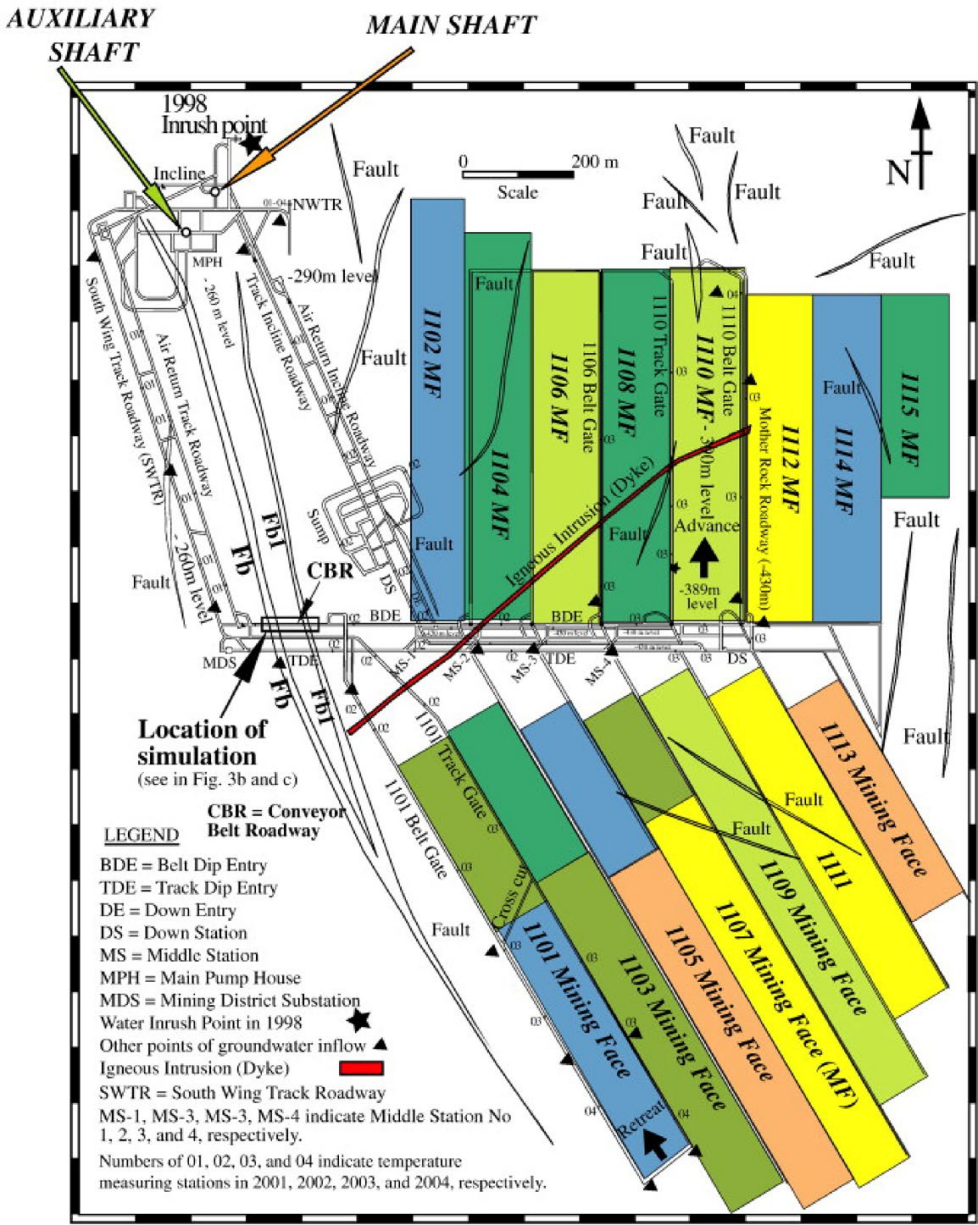
Layout of longwall mining panels in the Barapukuria Coal Mine, Bangladesh^42^.

In the Barapukuria coal basin, six coal seams have been identified; however, only Seam VI, with an average thickness of approximately 36 m, has been selected for mining operations. While single-slice or double-slice extraction is effective for seams with thicknesses in the range of 3–10 m as commonly practiced in countries such as China, the United States, Germany, Australia, the United Kingdom, and India. It is economically unviable for Barapukuria due to the greater seam thickness. As a result, the China National Machinery Import and Export Corporation (CMC) adopted a multi-slice longwall mining strategy, dividing Seam VI into 16 panels, each further subdivided into 6 extraction slices (see Fig. 3).

**Fig. 4:**
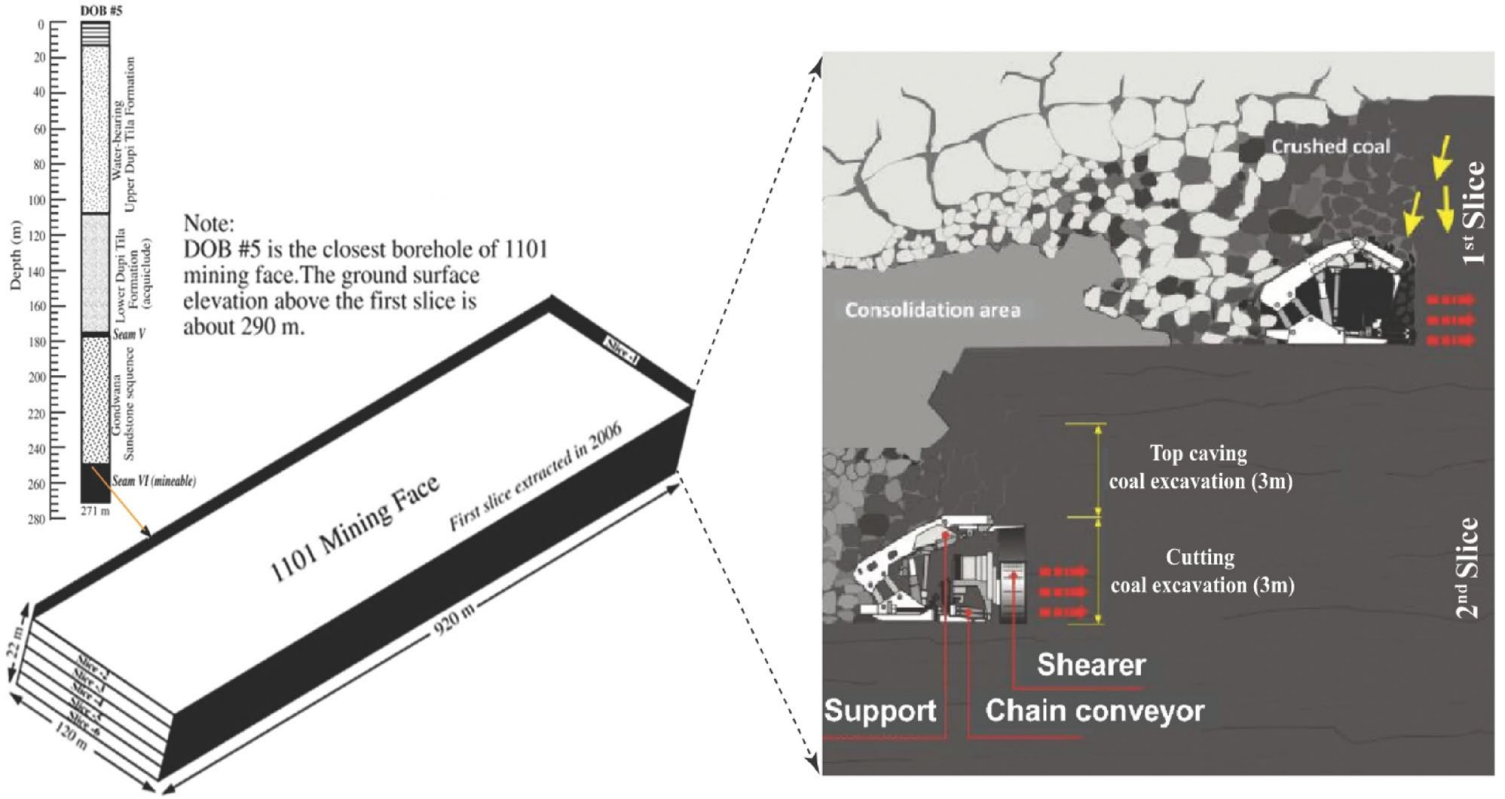
Schematic illustration of the multi-slice longwall top coal caving method, depicting sequential coal slice extraction and controlled overburden caving (modified from^43,44^).

Seam VI has been sectioned into panels with average dimensions of approximately 120 m in width, 550 to 900 m in length, and around 3.5 m in mining height, achieved through the development of perimeter roadways. For a panel height of 3.5 m, the resultant fracture zone is projected to extend up to 22 m above the mined-out area. Coal extraction from the first slice commenced in 2005 and has since continued under challenging geotechnical conditions, including elevated temperatures, high humidity levels, extensive roof caving, and significant water inflow from the overlying strata^43^. At present, a hybrid method combining multi-slice and LTCC is employed at the site. This approach has been gradually refined and optimized to address the substantial seam thickness, depth, and complex geo-mechanical conditions specific to the Barapukuria coal basin (see Fig. 4).

As of June 2024, a cumulative total of approximately 14.697 (million tons) Mt of coal has been extracted from the central zone of the Barapukuria Coal Mine (see Table 1).

**Table 1:** Coal extraction by slice and number of panels excavated^9^.

Slices	Number of panels	Coal extraction (Mt)
1st	13	4.338
2nd	10	6.451
3rd	6	3.625
4th	1	0.283

This total comprises 4.338 Mt extracted from the first slice, 6.451 Mt from the second slice, 3.625 Mt from the third slice, and 0.283 Mt from the fourth slice. The first slice involved the excavation of 13 panels, followed by 10 panels in the second slice, 6 panels in the third, and a single panel in the fourth slice-collectively contributing to the mine’s total coal production.

## Numerical model based on FDM

In the field of geo-mechanical and mining engineering simulations, widely utilized numerical modelling software includes FLAC, UDEC, ANSYS, and ADINA. For this study, FLAC3D version 7.0, developed by ITASCA (USA) and released on February 26, 2024, was selected as the primary simulation tool.

### Numerical model established and input parameters

**Fig. 5:**
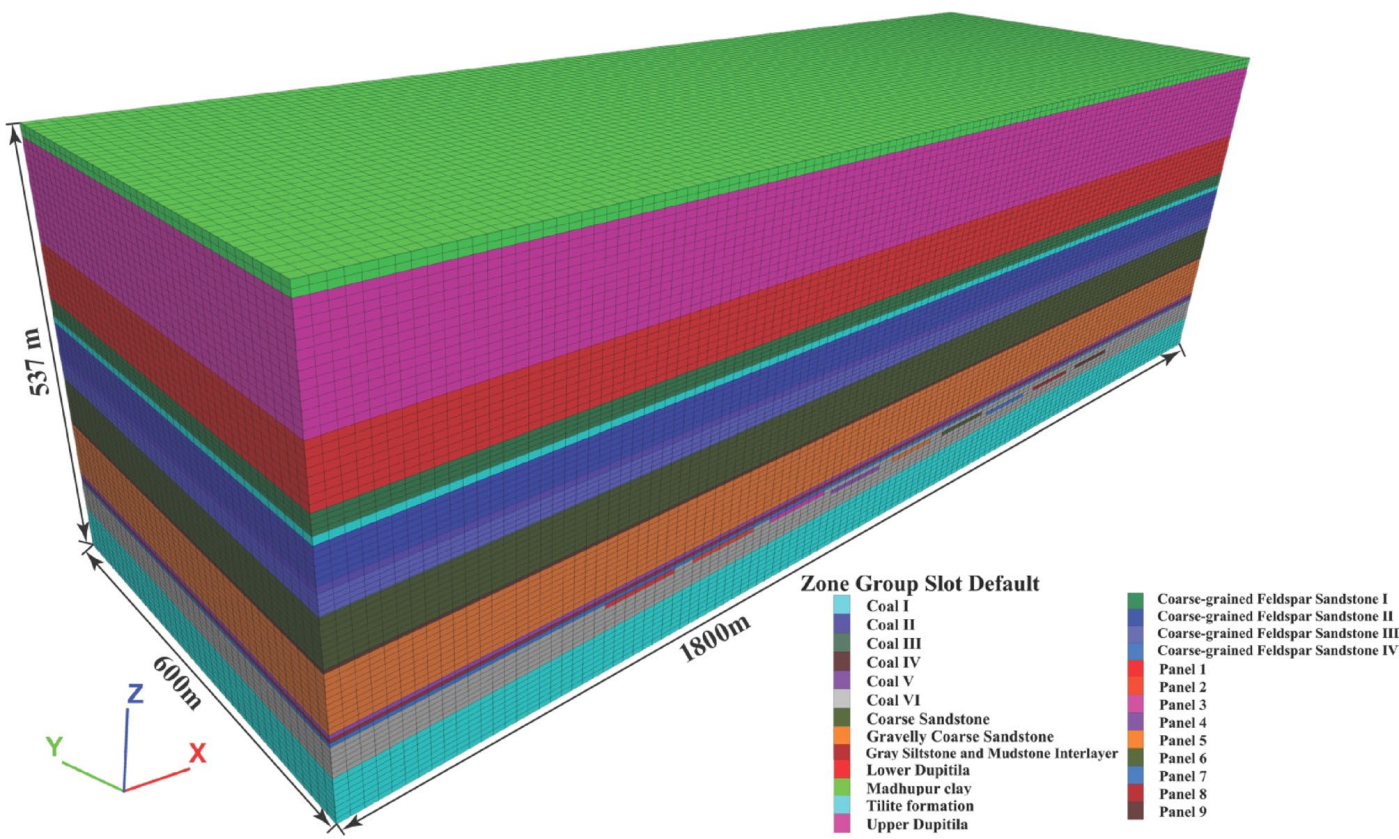
Geometric configuration of the numerical model used in the simulation.

In the numerical simulation, the model boundaries are primarily defined based on the spatial extent of the mining-induced disturbance zone. Reflecting actual subsidence conditions, the model dimensions are set to 1800 m along the X-axis (perpendicular to the mining direction), 600 m along the Y-axis (parallel to the mining direction), and 537 m along the Z-axis (vertical direction). To balance computational efficiency with simulation accuracy, the model adopts real-scale dimensions. The horizontal mesh resolution is 15 m, while the vertical resolution varies between 5 and 10 m, depending on stratigraphic complexity. The constructed model comprises 364,800 zones and 381,997 grid nodes, as illustrated in Fig. 5.

The constructed mesh model achieves a balance between computational efficiency and simulation accuracy. Given that this study emphasizes surface subsidence within the mining zone, finer zoning was applied to enhance the resolution of surface deformation analysis. Mesh generation followed a stratified refinement approach: in the vertical direction, zone dimensions decrease progressively toward the surface to capture detailed subsidence behaviour. In sedimentary formations, where lithological layering is often irregular and some strata are comparatively thin due to depositional variability, incorporating every individual layer into the FLAC3D model would substantially increase computational demand and compromise modelling feasibility. Consequently, a selective stratification approach was adopted. This approach integrates geological design requirements with the physical and mechanical characteristics of the rock mass to optimize model simplification. The corresponding numerical simulation parameters used in FLAC3D are summarized in Table 2.

**Table 2:** Geo-mechanical properties.

Rock stratum	Strata thickness (m)	Bulk modulus (MPa)	Shear modulus (MPa)	Friction angle $(\varphi$)	Cohesion (MPa)	Density (kg/m^3^)	Tension (MPa)
Madhupur clay	15	12.670	7.6000	15	1.10	1200	0.18
UDT	120	13.100	9.0200	23	1.11	1230	0.17
LDT	67	315.56	236.67	26	1.12	1470	0.23
Coarse-grained Feldspar Sandstone I	22	478.40	315.04	35	2.00	2640	0.52
Coal I	9	278.43	244.83	20	1.62	1430	0.32
Coarse-grained Feldspar Sandstone II	40	478.40	315.04	35	2.50	2640	0.35
Coal II	8	278.92	245.26	20	2.50	1430	0.28
Coarse-grained Feldspar Sandstone III	23	287.36	206.61	31	2.40	2640	0.58
Coal III	2	336.27	295.69	20	2.50	1430	0.28
Coarse Sandstone	54	184.48	132.64	35	2.60	2640	0.49
Coal IV	5	286.76	252.16	20	2.50	1430	0.28
Gravelly Coarse Sandstone	68	200.00	137.70	37	3.00	2680	0.25
Coal V	5	276.96	243.53	20	2.51	1430	0.28
Gray Siltstone and Mudstone Interlayer	4	149.38	98.370	33	2.71	2600	0.25
Coarse-grained Feldspar Sandstone IV	5	126.44	90.910	31	2.00	2500	0.25
Coal VI	36	368.14	323.71	20	3.20	1430	0.60
Tilite formation	55	740.74	487.80	33	2.12	2600	0.63

The mining process was divided into three sequential phases to simulate progressive seam extraction. (a) Phase 1 involved the initial excavation of the first slicing level, encompassing panel 1, panel 2, and panel 3. (b) Phase 2 proceeded with the development of deeper segments of the seam, adding the second slicing level through panel 4, panel 5, and panel 6. (c) Phase 3 further extended the mining depth by incorporating the third slicing level, which included panel 7, panel 8, and panel 9.

**Fig. 6:**
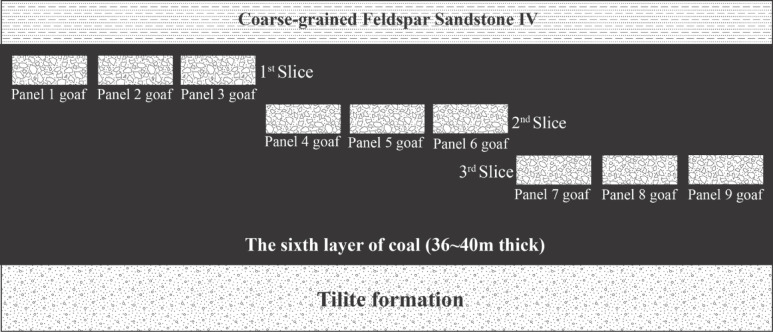
Schematic representation of the mined-out area distribution across multi-slicing phases.

This phased multi-slice mining strategy reflects a stepwise deepening of exploitation within the coal seam. The spatial distribution of the mined-out areas across the three slicing stages is illustrated in Fig. 6.

**Fig. 7:**
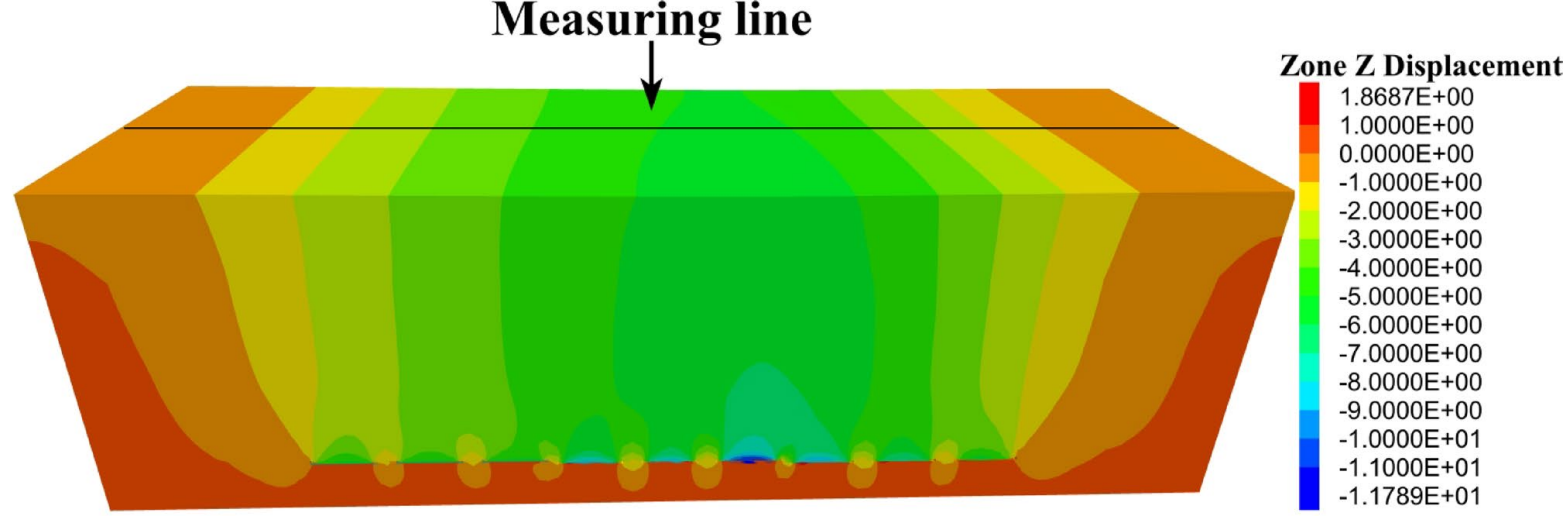
Configuration of measurement lines used for monitoring ground deformation in the numerical model.

To monitor ground deformation, a network of measurement lines was superimposed onto the numerical model, as illustrated in Fig. 7.

Observation points were uniformly distributed along each line at 15 m intervals, enabling systematic collection of displacement data throughout the simulation process. This configuration facilitated a detailed and accurate assessment of surface subsidence characteristics induced by progressive multi-slice mining.

### The constitutive mechanical model and yield criterion

This study employs an incremental elastoplastic constitutive model in conjunction with the Mohr–Coulomb failure criterion, incorporating a tension cutoff to capture tensile failure behaviour. The failure conditions for each element are defined as follows:

When the element is compressive:

1 $$\sigma_{1} - \frac{1 + \sin \varphi }{{1 - \sin \varphi }}\sigma_{3} - 2c\sqrt {\frac{1 + \sin \varphi }{{1 - \sin \varphi }}} = 0$$

When the element is tensile:

2 $${\upsigma }_{1}-{\upsigma }_{t}=0$$

where ${\upsigma }_{1}$ is the maximum principal stress, MPa; ${\upsigma }_{3}$ is the minimum principal stress, MPa; $\varphi$ is the internal friction angle, °; $c$ is the cohesion, MPa; ${\upsigma }_{t}$ is the tensile strength, MPa.

### Boundary conditions and the initial state of stress

To minimize boundary effects and ensure that mining-induced displacements did not influence model edges, a sufficiently large model domain was adopted. The boundary conditions were defined as follows: (1) Displacement constraints in the X-direction were applied to the eastern and western boundaries; (2) Constraints in the Y-direction were imposed on the southern and northern boundaries; (3) The Z-direction (vertical) was constrained at the model base. No constraints were applied to the top surface, which was modelled as a free face to allow for realistic deformation responses to mining activities. Displacements at all four lateral boundaries and the bottom boundary were fixed at zero in their respective normal directions. The model was subjected solely to gravitational loading.

**Fig. 8:**
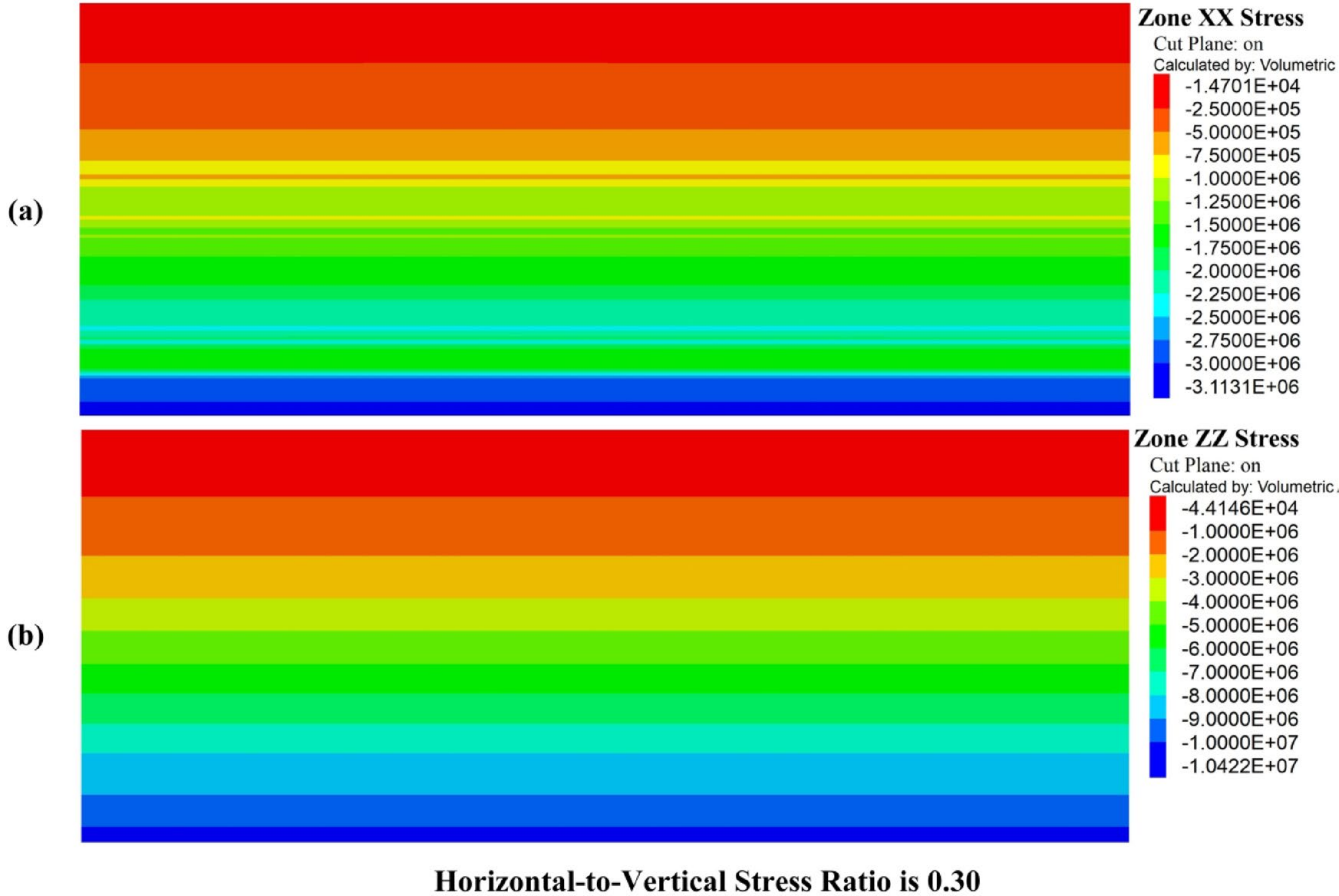
Stress distribution in the surrounding rock during mining phases. (**a**) Horizontal stress (Zone XX) contours. (**b**) Vertical stress (Zone ZZ) contours**.**

Figure 8a shows the vertical stress contours at various mining phases, highlighting a significant concentration of vertical stress around the mined-out zones. This observation indicates that vertical stresses play a dominant role in subsidence, particularly as mining depth increases. The intensity of vertical stress around the mined areas contributes to surface deformation, suggesting that vertical compaction mechanisms are the primary drivers of subsidence. The redistribution of vertical stress in these zones is crucial for understanding how surface deformations manifest during different mining phases.

In contrast, Fig. 8b presents the horizontal stress contours, which are more uniformly distributed across the modelled area. The horizontal stress does not show the same concentration observed in the vertical stress contours. The horizontal-to-vertical stress ratio in the model was calculated to be 0.30, meaning that horizontal stresses are less influential compared to vertical stresses in the surrounding strata. This further supports the conclusion that vertical stresses dominate the surface deformation process, while horizontal stresses play a secondary role, particularly in the deeper mining phases.

### Numerical simulation results of mining overburden deformation

#### Stresses analysis

**Fig. 9:**
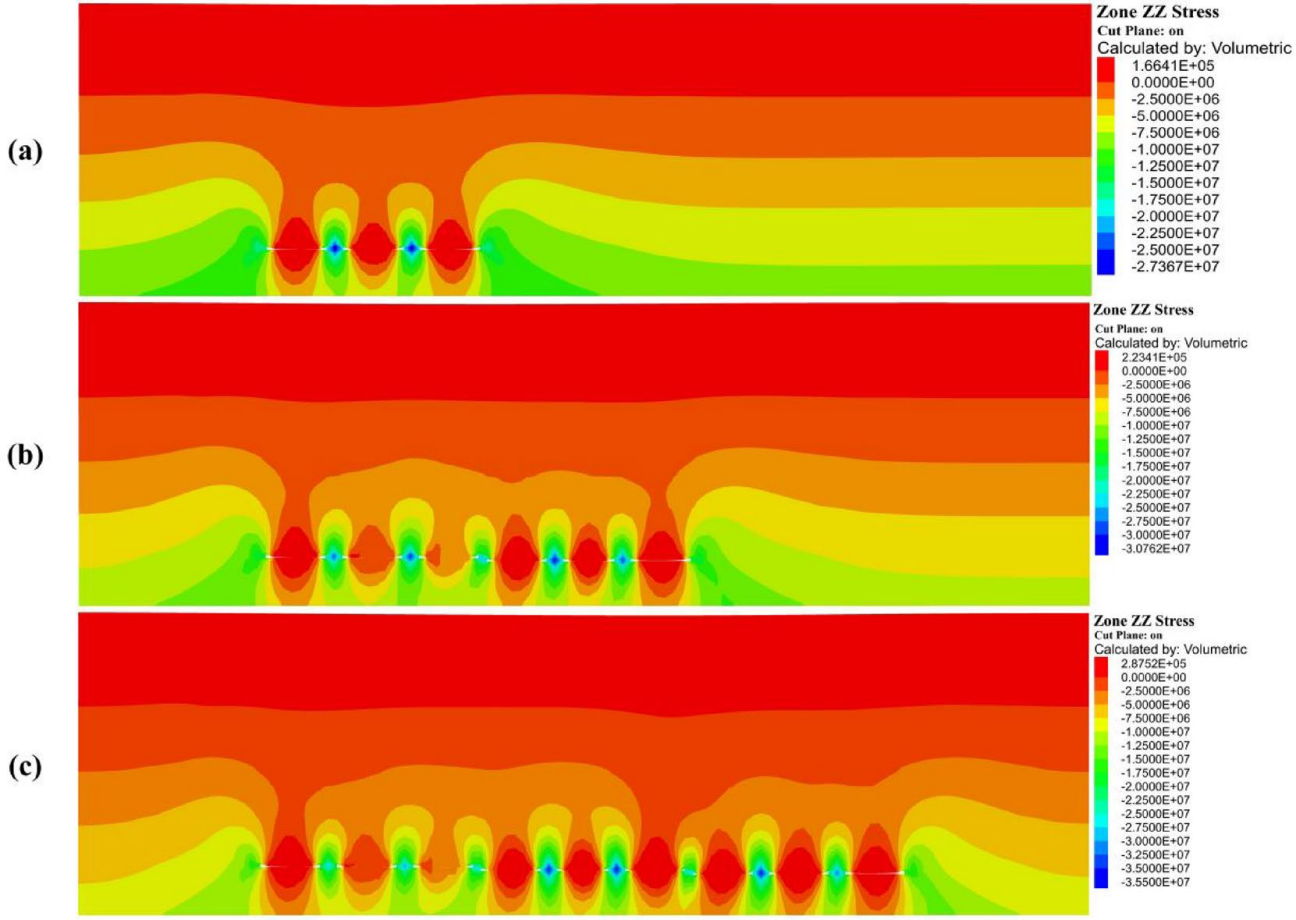
Vertical stress distribution in the surrounding rock at different mining slices: (**a**) First slice, (**b**) Second slice, and (**c**) Third slice.

**Fig. 10:**
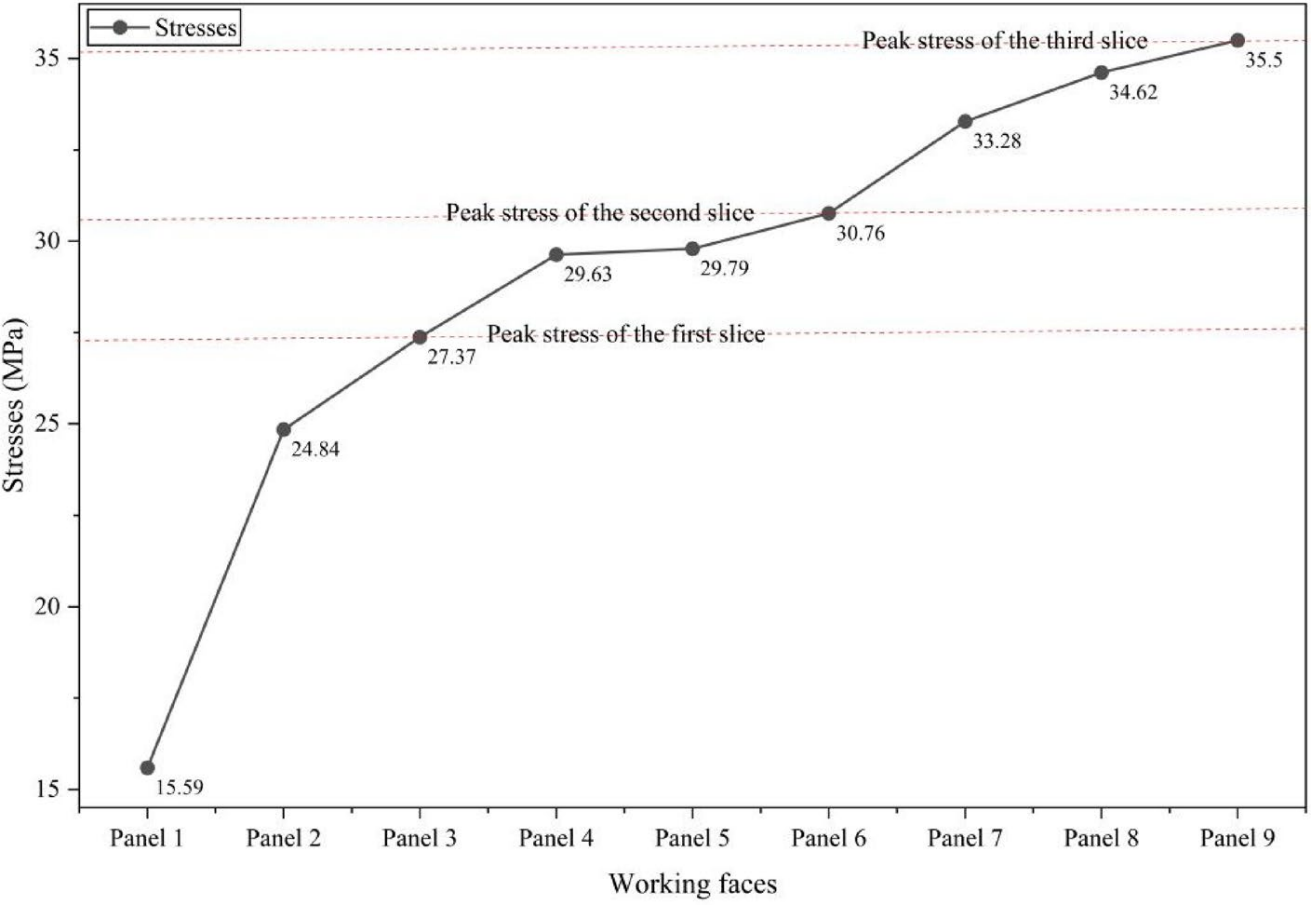
Curve illustrating the distribution of vertical stress across the mined panels.

Excavation of an underground opening within a pre-stressed rock mass leads to a redistribution of the in-situ stress field, primarily due to the removal of confining material^45^. In this study, stress evolution was assessed following the sequential excavation of three mining slices, simulating the progressive extraction characteristic of multi-slice longwall mining. It is important to note that these “slices” are conceptual abstractions within the numerical model, intended to represent phased mining at increasing depths within the coal seam. The resulting stress fields reflect the post-excavation equilibrium states and are used to analyse the cumulative geo-mechanical response of the rock mass to multi-seam extraction, rather than transient stress conditions during active mining. Figure 9 displays the vertical stress contours generated by FLAC3D for each mining phase, while Fig. 10 provides a line chart showing the peak vertical stress values recorded above each panel, offering a quantitative representation of stress accumulation throughout the entire multi-slice sequence.

As longwall panel excavation progressed, significant redistribution of vertical stress was observed within the overburden. In Phase 1, corresponding to the extraction of Panels 1 to 3 (Fig. 9a), vertical stress concentrations emerged along the peripheries of the mined-out voids. The stress contours revealed the formation of localized abutment pressure zones adjacent to the excavation boundaries, accompanied by stress arching above the goaf. Stress relief was concentrated within the caved regions, while peak vertical stress values were recorded near Panel 3 at 27.37 MPa. Maximum stresses for Panels 1 and 2 were 15.59 MPa and 24.84 MPa, respectively. This distribution indicates a rising stress gradient from left to right, likely influenced by the dip of the coal seam and asymmetrical in-situ geological conditions.

In Phase 2 (Fig. 9b), corresponding to the extraction of Panels 4 through 6, the overburden exhibited intensified stress interactions between previously mined and currently active slices. Abutment stress zones became more prominent and began to overlap, particularly in the central region, resulting in the development of a complex, multi-lobe stress field. The maximum vertical stress increased further, reaching 30.76 MPa near Panel 6, while Panels 4 and 5 recorded 29.63 and 29.79 MPa, respectively. These elevated stress values reflect the compounded load transfer associated with sequential seam extraction. Stress trajectories extended both deeper into the strata and laterally across the surrounding rock mass, indicating a widened influence zone adjacent to the mined-out areas.

In Phase 3 (Fig. 9c), corresponding to the extraction of Panels 7 through 9, the simulation revealed the most pronounced vertical stress accumulations. Peak stress values continued to rise, reaching 35.50 MPa at Panel 9, with Panels 7 and 8 recording 33.28 MPa and 34.62 MPa, respectively. The stress distribution also exhibited greater asymmetry, likely influenced by geological heterogeneity or variations in seam geometry. Additionally, the development of extensive low-stress zones above the goaf indicated progressive unloading and the lateral migration of stress into adjacent undisturbed rock masses. This stage reflects a mature state of stress redistribution, marked by elevated abutment loading and increased potential for instability in unsupported areas. Collectively, these findings demonstrate that multi-slice longwall mining induces nonlinear and cumulative alterations to the stress regime—critical considerations for effective strata control and longwall layout design.

The progressive redistribution of vertical stress induced by multi-slice longwall mining is a key driver in the initiation and evolution of surface subsidence. As excavation proceeds, the removal of support within the mined-out zones results in the transfer of overburden loads to adjacent unmined regions and deeper strata. This redistribution process leads to stress relief within the goaf, accompanied by heightened stress concentrations in the surrounding rock mass, thereby compromising the structural integrity of the overlying strata. As a consequence, the overburden undergoes bending, fracture propagation, and compaction—processes that manifest as measurable surface subsidence. The intensity and spatial extent of this deformation are directly governed by the magnitude and pattern of stress redistribution. Thus, a comprehensive understanding of stress transfer mechanisms is critical not only for accurate subsidence prediction but also for the development of effective control measures aimed at safeguarding surface infrastructure and preserving environmental stability.

#### Analysis of overlying strata deformation characteristics

**Fig. 11:**
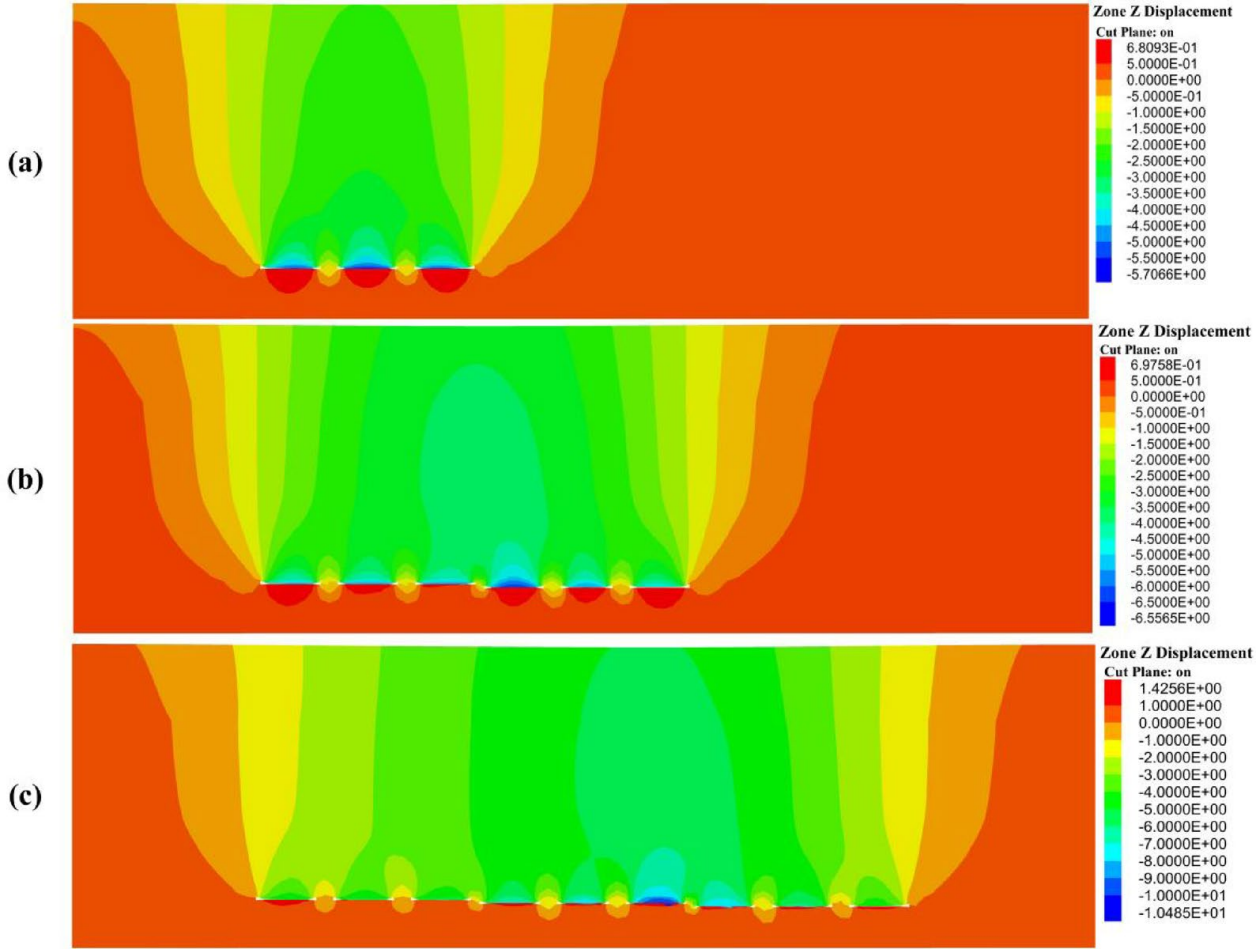
Vertical displacement of the overlying strata at different mining slices: (**a**) First slice, (**b**) Second slice, and (**c**) Third slice.

Figure 11 illustrates the vertical displacement evolution of the overlying strata under sequential multi-slice extraction. The maximum displacements recorded above each panel are detailed in the subsequent discussion. The results reveal a clear trend of progressively increasing deformation, both in magnitude and spatial extent, corresponding with each successive mining slice.

In Phase 1 (Fig. 11a), the simulation captured the initial formation of subsidence troughs above the mined-out voids. Maximum vertical displacements were recorded at 4.02 m, 5.09 m, and 5.71 m above Panels 1, 2, and 3, respectively. The displacement contours exhibited notable asymmetry, with a broader zone of influence extending to the left side of the goaf. Subsidence extended significantly beyond the panel boundaries, indicating that the overburden response is not strictly limited to the immediate mining area. These observations imply that factors such as geological dip and stratigraphic heterogeneity may contribute to the uneven spatial distribution of surface settlement.

In Phase 2 (Fig. 11b), the cumulative impact of sequential slice extraction became increasingly evident. Maximum vertical displacements rose to 6.11 m, 6.12 m, and 6.56 m above Panels 4, 5, and 6, respectively. Compared to Phase 1, the subsidence troughs deepened and expanded laterally, with adjacent deformation zones beginning to coalesce into a continuous subsidence basin. This pattern reflects the superimposed deformation of the overburden, wherein compaction and flexural bending induced by earlier slices were exacerbated by subsequent extractions. Notably, the central panels experienced the most significant vertical displacement, underscoring the compounded effects of load transfer and stress redistribution within the strata.

In Phase 3 (Fig. 11c), subsidence reached its most advanced stage. Maximum vertical displacements of 7.69 m, 9.43 m, and 10.49 m were observed above Panels 7, 8, and 9, respectively—marking the peak deformation throughout the entire mining sequence. The subsidence troughs developed into a wide, continuous basin, characterized by pronounced overburden bending, fracturing, and compaction. Asymmetry in the subsidence profile became more evident, with one side exhibiting steeper settlement gradients. These findings confirm that multi-slice longwall mining induces a nonlinear and cumulative subsidence response, wherein deeper extractions exert a substantially greater influence on surface deformation.

#### Analysis of numerical simulation results of surface subsidence

**Fig. 12:**
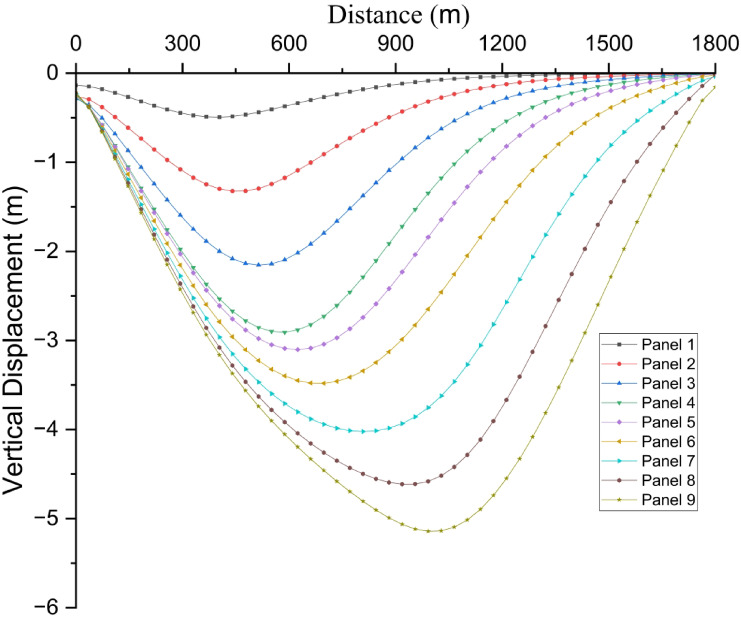
Surface subsidence profiles corresponding to different panels of multi-slice longwall mining.

**Fig. 13:**
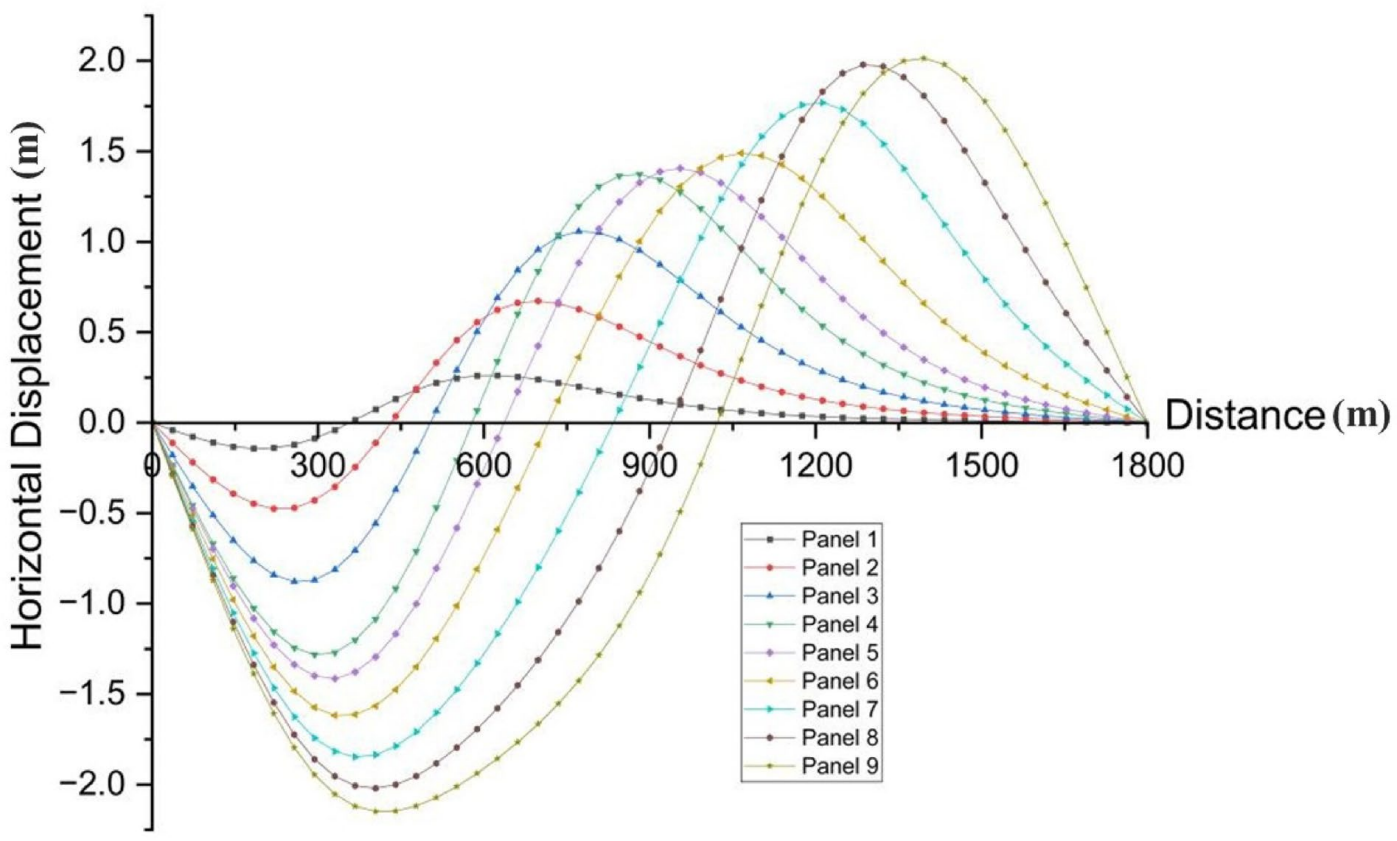
Curves illustrating surface tilt and lateral ground movement induced by multi-slice mining.

Surface deformation remains a fundamental issue in underground coal mining, particularly in assessing risks to surface infrastructure, transportation networks, and surrounding environmental systems^46^. Multi-slice longwall mining induces progressive collapse and compaction of the overburden strata, leading to cumulative surface subsidence and significant horizontal ground deformation, as a result of sequential extraction without structural support in the goaf^47^. This section provides a comprehensive analysis of surface deformation characteristics resulting from multi-slice longwall extraction, based on numerical simulation results aligned with the direction of mining advance. The corresponding surface subsidence and horizontal displacement profiles are illustrated in Fig. 12 and Fig. 13, respectively. Figure 12 illustrates the progression of maximum surface subsidence corresponding to each mining slice.

Subsidence deformation develops distinctly across the three mining stages. During the first slice (Panels 1–3), the observed maximum surface subsidence values are 0.49 m, 1.32 m, and 2.15 m, respectively. These displacements are attributed to the collapse of overburden into the goaf, initiating asymmetrical subsidence troughs that extend beyond the panel margins. In the second slice (Panels 4–6), subsidence increases markedly to 2.91 m, 3.10 m, and 3.48 m, reflecting intensified vertical movement due to reactivation and further compaction of previously disturbed strata. The resulting troughs deepen and laterally merge, forming a wider subsidence basin. The third slice (Panels 7–9) exhibits peak subsidence values of 4.02 m, 4.61 m, and 5.14 m, representing the maximum vertical deformation across the entire mining sequence. This extensive settlement is driven by cumulative compaction and progressive bending of the overlying strata under successive load transfers induced by multi-slice extraction. The observed trend clearly demonstrates that with continued slicing, the structural integrity of the overburden deteriorates across an expanded area, leading to both greater magnitude and spatial extent of surface subsidence. In contrast to backfill mining—where voids are partially filled and roof contact restricts collapse—the caving method permits uncontrolled vertical deformation due to the absence of support. Beyond vertical settlement, horizontal displacement is a key parameter in evaluating surface deformation. Figure 13 illustrates the maximum surface inclination (tilt) recorded throughout the mining progression.

During the first slice, horizontal movement is moderate but already noticeable, with displacements of 0.26 m, 0.67 m, and 1.06 m observed in Panels 1 through 3. These movements result from lateral stretching of the overburden as roof collapse initiates and surrounding strata adjust. As extraction advances into the second slice (Panels 4–6), horizontal deformation intensifies, reaching 1.37 m, 1.41 m, and 1.49 m, respectively. The expansion and deepening of subsidence troughs amplify lateral strain and shear deformation within the overlying layers. In the third slice (Panels 7–9), horizontal displacements peak at 1.65 m, 1.81 m, and 1.91 m, representing the highest deformation magnitudes across the mining sequence. These values reflect the combined effect of enlarged voids and progressive roof collapse, which induce substantial tensile stress and stretching in the upper strata. The continual increase in horizontal displacement underscores the influence of unsupported deep seam extraction, where the absence of backfill contributes to significant lateral ground movement. Such deformation elevates the likelihood of surface cracking, tilt-induced structural instability, and slope failures. A comprehensive summary of surface deformation metrics for all mining stages is provided in Table 3.

**Table 3:** Maximum surface deformation at multi slicing mining.

Phases	Panels	Maximum value of ground subsidence (m)	Maximum value of horizontal displacement (m)	Subsidence ratio	Horizontal co-efficient
1st slice	Panel 1	0.49	0.26	0.08	0.53
	Panel 2	1.32	0.67	0.22	0.51
	Panel 3	2.15	1.06	0.36	0.49
2nd slice	Panel 4	2.91	1.37	0.49	0.47
	Panel 5	3.10	1.41	0.52	0.45
	Panel 6	3.48	1.49	0.58	0.43
3rd slice	Panel 7	4.02	1.65	0.67	0.41
	Panel 8	4.61	1.81	0.77	0.39
	Panel 9	5.14	1.91	0.86	0.37

**Fig. 14:**
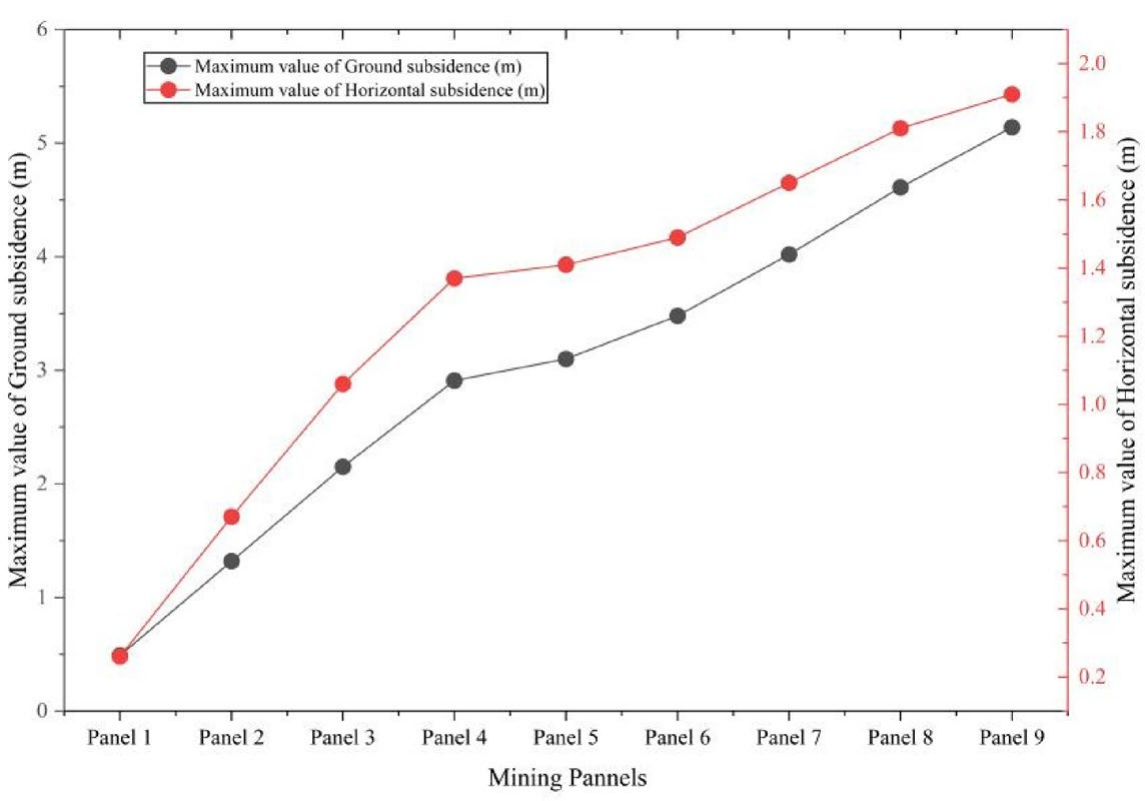
Correlation between maximum ground subsidence and horizontal displacement.

These results exhibit a distinct nonlinear escalation in surface deformation with increasing mining depth. Both vertical subsidence and horizontal displacement intensify substantially from the first to the third mining slice. Notably, the subsidence ratio—defined as the ratio of vertical displacement to mining thickness—increases from 0.08 to 0.86, reflecting the growing influence of vertical compaction. Conversely, the horizontal deformation coefficient declines from 0.53 to 0.37, suggesting a relative reduction in lateral strain. This trend highlights the predominance of vertical compaction mechanisms over horizontal displacement in deeper mining stages. Figure 14 illustrates the correlation between maximum vertical subsidence and the corresponding horizontal displacement across all nine panels.

**Fig. 15:**
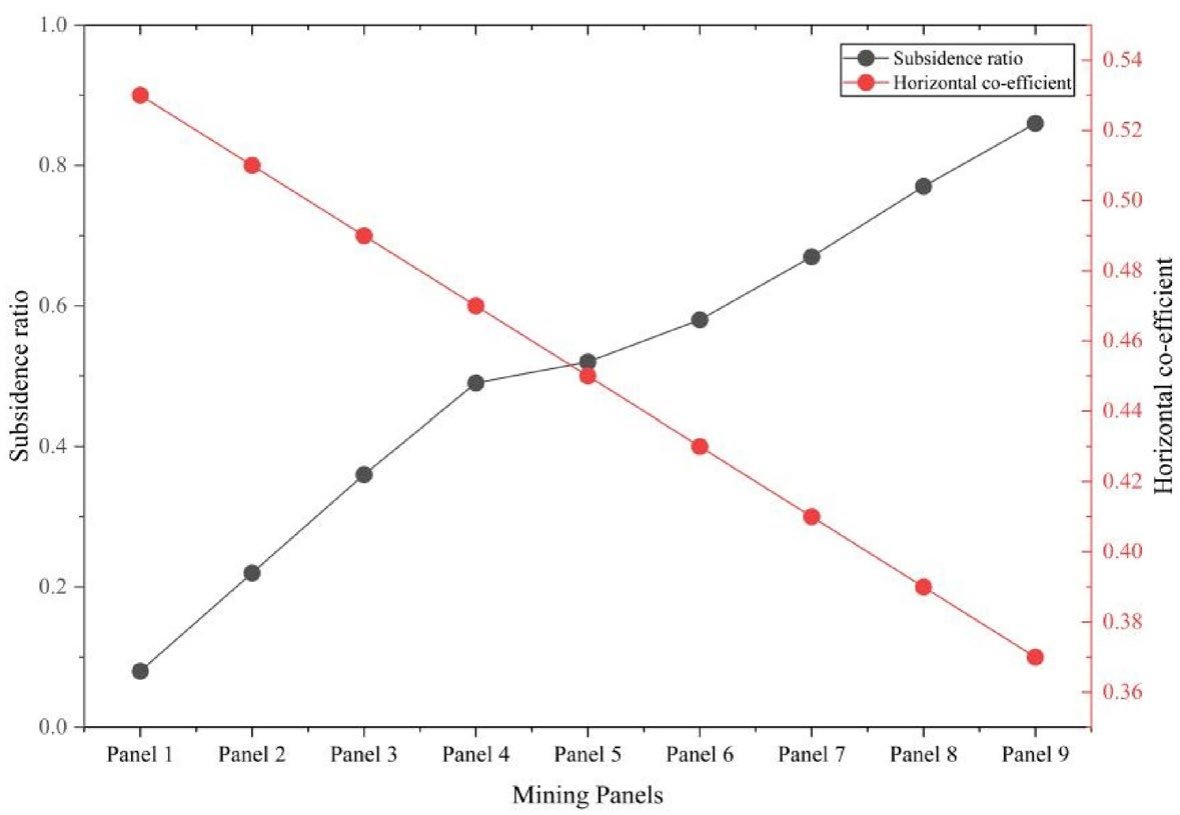
Relation between subsidence ratio and horizontal co-efficient.

The data exhibit a clear upward trend, indicating that greater subsidence is consistently associated with increased horizontal movement. This relationship emphasizes the three-dimensional interactive response of the overburden, wherein vertical collapse induces lateral extension through bending deformation and interlayer shear. The coupling between vertical and horizontal deformation is a critical consideration for the development of accurate subsidence prediction models, surface hazard assessments, and the design of infrastructure within mining-affected areas. Figure 15 depicts the relationship between the subsidence ratio and the horizontal coefficient across successive mining stages.

As mining advances, the subsidence ratio increases due to the accumulation of vertical deformation, whereas the horizontal coefficient exhibits a declining trend. This indicates that although the overall magnitude of ground movement rises, the proportion of lateral displacement relative to vertical settlement diminishes in deeper extractions. Specifically, the subsidence ratio increases from 0.08 in Panel 1 to 0.86 in Panel 9, while the horizontal coefficient decreases from 0.53 to 0.37. This inverse correlation arises from the progressive compaction of the overburden, which restricts horizontal expansion at greater depths. These findings offer important insights into subsidence mechanisms and aid in delineating potential surface failure zones for risk evaluation and mitigation.

The progressive intensification of surface deformation with increasing depth in multi-slice longwall mining is clearly reflected across all evaluated parameters—subsidence, surface inclination, and deformation ratios. In contrast to backfill-supported systems, longwall caving generates extensive goaf areas, resulting in higher magnitudes of vertical subsidence, more pronounced horizontal ground displacement, and a decreasing ratio of horizontal to vertical deformation at greater depths. These outcomes underscore the critical role of numerical simulation in optimizing longwall mining design and emphasize the necessity of implementing effective subsidence control strategies to mitigate risks to surface infrastructure, particularly in densely populated or environmentally sensitive regions.

## Limitations and future research

The findings of this study on surface subsidence induced by multi-slice longwall mining in the Barapukuria coal basin show strong alignment with existing research, demonstrating the legitimacy of the approach. An earlier study on subsidence in the Barapukuria mining area reported maximum displacements of 5.8 m in the North-Western zone and 4.6 m in the North-Eastern zone, with the southern part experiencing up to 4.2 m of subsidence^3^. Similarly, this study observes comparable subsidence magnitudes, with the maximum surface displacement in the deeper mining phases reaching 5.14 m. The subsidence range in both studies (0.01 to 10.5 m) supports the credibility of the current research, confirming that the numerical modelling approach used here is consistent with the field data. The close agreement between the observed and simulated results highlights the validity of the model and affirms that the study’s findings are relevant and applicable to the Barapukuria coal basin.

However, some limitations should be acknowledged. A major limitation of this study is the reliance on available geological and rock mechanics data for the simulation. While the study incorporated detailed geological data, certain local variations, such as faults, fractures, and dykes, were excluded from the model for simplicity. These geological features can significantly influence subsidence patterns, and their exclusion may lead to discrepancies between predicted and observed surface deformations, particularly in regions with complex geology. Recent studies have highlighted the importance of considering these features when modeling subsidence^48–50^ . Future research could address this limitation by integrating these geological complexities into the model to enhance the accuracy of subsidence predictions in such areas.

Another limitation is the focus on short-term surface deformation, without considering long-term environmental factors like groundwater fluctuations, seasonal changes, or climatic conditions. These factors could influence subsidence behaviour over time, and their integration into future models would provide a more comprehensive understanding of the long-term sustainability of mining operations. Additionally, the study’s findings are specific to the Barapukuria coal basin, and while the results are consistent with existing data from the region, their applicability to other mining areas with different geological conditions requires further investigation. Extending this research to other regions with varying coal seam depths, geological formations, and mining methods would improve the generalizability of the findings.

Future research should focus on integrating real-time monitoring data from advanced technologies such as Ground-Penetrating Radar (GPR), Satellite-Based Interferometry (InSAR), and GPS displacement systems to enhance the accuracy of subsidence predictions. Combining these data sources with numerical simulations will provide a more precise and dynamic assessment of surface deformations. Additionally, incorporating Machine Learning (ML) and Deep Learning (DL) techniques into subsidence modelling holds great promise. ML algorithms like Support Vector Machines (SVM) and Random Forests could identify patterns in historical data and predict future subsidence trends. Deep learning models: e.g., Convolutional Neural Networks (CNNs), Recurrent Neural Networks (RNNs) could be used for analysing time-series data, improving real-time subsidence forecasts^10,51–53^.

Hybrid models combining ML with traditional numerical simulations could address limitations of individual methods, optimizing geological and mechanical parameters for more accurate predictions. These advancements will also assist in evaluating the broader impacts of subsidence on infrastructure and the environment, and in predicting long-term stability and rehabilitation needs for subsided areas. By integrating ML, DL, and real-time monitoring, future research can revolutionize subsidence prediction, enabling more accurate, real-time assessments and the development of data-driven strategies to mitigate environmental and structural impacts.

## Conclusions

This study investigates surface subsidence induced by multi-slice longwall mining in the Barapukuria coal basin, using FLAC3D simulations to model the mining-induced deformations. The results demonstrate a progressive, nonlinear increase in both vertical and horizontal surface displacements as mining advances through successive slices. The vertical subsidence, for example, ranged from 0.49 to 5.14 m, while horizontal displacements increased from 0.26 to 1.91 m as mining depth increased.

The study also quantified the subsidence ratio, which escalated from 0.08 in the first phase to 0.86 in the third. Simultaneously, the horizontal deformation coefficient decreased, indicating that vertical compaction mechanisms became more dominant with increasing mining depth. These findings highlight the cumulative nature of surface subsidence and the growing severity of deformation as mining progresses.

Furthermore, stress redistribution analysis revealed significant stress concentrations around the mined-out zones, with maximum vertical stress reaching 35.50 MPa in the third mining phase. This redistribution of stress contributed to increased compaction and fracturing of the overlying strata, which in turn intensified surface subsidence. These insights emphasize the importance of understanding stress transfer mechanisms to improve subsidence prediction models.

In conclusion, the study underlines the need for more advanced subsidence prediction models and effective mitigation strategies. These measures are particularly crucial in regions with dense infrastructure or environmental sensitivity. Integrating stress redistribution mechanisms into these models is essential to better manage the long-term impacts of mining activities on the surface and reduce risks to both infrastructure and the environment.

## Data Availability

The data used to support the findings of this study are included in the article.

## Abbreviations

$\varphi$: Internal friction angle
$${\sigma }_{1},$$$${\sigma }_{3}$$: Maximum and minimum principal stresses
$$c$$: Cohesion
$${\sigma }_{t}$$: Tensile strength
*MPa*: Mega pascal
*Mt*: Million tonnes
*m*: Meters
*Km*^*2*^: Square kilometres
*N*: North
*S*: South
*W*: West
*E*: East
BC: Basement complex
CNN: Convolutional neural network
CMC: China national machinery import and export corporation
DL: Deep learning
DT: Dupi Tila
EBF: Eastern boundary fault
FDM: Finite difference method
FEM: Finite element method
GIS: Geographic information systems
GPR: Ground-penetrating radar
GW: Gondwana formation
InSAR: Satellite-based interferometry
LDT: Lower Dupi Tila formation
LTCC: Longwall top coal caving
MC: Madhupur clay formation
ML: Machine learning
RNN: Recurrent neural network
SVM: Support vector machines
DEM: Distinct element method
UDT: Upper Dupi Tila formation
